# Clinical Outcomes Following Use of a Next-Generation Cellular Bone Matrix in High-Risk Patients Undergoing Arthrodesis: A Retrospective Analysis

**DOI:** 10.7759/cureus.114072

**Published:** 2026-08-06

**Authors:** Kurt J Hofmann, Colin Wood, Francesca Totis, Peter G Passias

**Affiliations:** 1 Department of Orthopedic Surgery, Boston Bone and Joint Institute, Waltham, USA; 2 Department of Orthopedic Surgery, New England Baptist Hospital, Boston, USA; 3 Spine Division, Departments of Orthopedic and Neurological Surgery, Duke University Health System, Durham, USA

**Keywords:** biologics, bone graft, bone graft substitute, cellular bone matrix, demineralized bone graft, foot and ankle arthrodesis, foot and ankle fusion, regenerative medicine, spinal fusion, spine arthrodesis

## Abstract

Background

Arthrodesis is a standard intervention for musculoskeletal pain, yet non-union remains a significant challenge. Systemic comorbidities such as smoking, diabetes, and obesity can lead to biological deficiencies in the signaling proteins required for bone healing at the surgical site. This study evaluates the clinical safety and efficacy of a next-generation cellular bone matrix (CBM) designed to preserve high concentrations of endogenous growth factors and viable cells that may help address potential biological deficits in at-risk populations.

Methods

Data were pooled from two single-center retrospective studies. The analysis included 35 high-risk participants (mean age 61.4 years with an average of 2.6 risk factors per participant) undergoing either spinal (n=18) or foot and ankle arthrodesis (n=17). Risk factors included advanced age, tobacco use, diabetes, obesity, hypertension, and revision surgery, among others. Primary efficacy was defined as radiographic fusion at standard follow-up time points. Rates of major complications and unplanned revisions were examined for safety.

Results

Despite these high-risk profiles, radiographic union rates were high in both cohorts. In the spine group, 100% fusion was achieved for all interbody (n=33/33) and posterolateral (n=95/95) levels by 12 months, with a median time to fusion of 6.0 months. Notably, the time to fusion was not significantly impacted by construct length in this small, exploratory subgroup comparison.

In the foot and ankle cohort, 100% radiographic fusion was achieved by 24 weeks, with a median time to fusion of 6.0 weeks. There were no statistically significant differences in healing times between forefoot/midfoot and hindfoot/ankle subgroups, though these subgroups were also small and likely underpowered. Across both cohorts, the rate of non-union and graft-related adverse events was 0% (n=0).

Conclusions

In this small retrospective study, use of the next-generation CBM was associated with favorable fusion rates and no reported graft-related complications in a high-risk patient population. These findings are consistent with the hypothesis that a concentrated source of native growth factors and viable cells may support fusion even in biologically compromised environments. However, future powered studies, including a control group and larger patient population, are needed to confirm our preliminary findings and determine causation.

## Introduction

Arthrodesis is a standard surgical intervention performed to alleviate chronic pain and restore function in both the axial and appendicular skeleton. However, the risk of non-union -- a complication occurring in up to 35% of lumbar spine and 41% of foot and ankle cases -- remains a significant clinical challenge [1-3]. This is especially problematic, as the rate of procedures has significantly increased over time in patients 65 years of age and older who have existing comorbidities and significant risk factors for non-union [4-6]. Complications from non-union can lead to prolonged disability and/or revision surgery and contribute to increased healthcare costs [7,8].

Successful bone fusion requires a balance of biological and mechanical factors [9]. Systemic risk factors such as tobacco use, vascular disease, chronic conditions such as hypertension and obesity, and metabolic issues such as renal disease and diabetes can negatively affect the local healing environment [6,8,10]. Even common medications such as non-steroidal anti-inflammatory drugs and steroids can interrupt important signaling during bone repair [10,11]. 

In patients presenting with one or more of these risk factors, the local biological environment at the surgical site is often deficient in critical components required for successful bone remodeling [8,11-13]. The ideal solution for bridging this deficiency is a complete graft comprised of osteoconductive, osteoinductive, and osteogenic components. Historically, autologous iliac crest bone graft was used, yet local autograft now serves as the preferred alternative. However, its limited volume and varying quality (especially in high-risk patients) are often inadequate for complex reconstructions [14,15].

The unmet need for bone graft substitutes that are capable of supporting bone healing without the limitations of autologous harvesting has led to the development of advanced cellular bone matrix (CBM) allografts enriched with endogenous growth factors and cells [8,16]. These proteins naturally recruit stem cells to an injured site and support differentiation into bone-forming osteoblasts [8,9,11]. In high-risk patients, the compromised biological environment could benefit from a complete set of bone healing supportive signals delivered from the graft [8,11].

Cellular allografts such as CBM are often rich in angiogenic and other soft tissue supporting growth factors that are vital for nutrient and cell transportation during bone healing [17]. However, traditional manufacturing often dilutes these inductive proteins within the bone matrix [18,19]. A next-generation CBM was developed to preserve a higher concentration of these native growth factors [20], including osteoinductive, chemotactic, angiogenic, and proliferative proteins, alongside functional, viable cells. This manuscript reports our clinical experience from using this novel CBM in high-risk patients undergoing foot, ankle, and spinal arthrodesis.

## Materials and methods

Study design

This study was a collaborative work between the participating institutes. Briefly, data from two single-center retrospective studies were pooled and analyzed to evaluate the clinical outcomes of a subset of participants at high risk for pseudoarthrosis. This combined subgroup analysis was performed post hoc and was not pre-specified in the protocol of either parent study. Patients included in the individual studies underwent either a spinal fusion or a foot and ankle fusion procedure using a novel CBM allograft prepared per manufacturer instructions (ISTO Biologics, Hopkinton, MA). Due to the retrospective nature of the study, patient- and lot-specific growth factor concentrations and cell viability for the grafts used in this cohort were not available for analysis. The Ethics Committee of each participating institution approved study data collection.

The total spinal fusion dataset consisted of 20 participants who received the CBM at 1 to 5 contiguous interbody levels and/or 1 to 15 contiguous posterolateral levels between November 2022 and May 2023. The foot and ankle dataset consisted of 20 participants who received the CBM alongside standard fixation in the ankle, hindfoot, midfoot, or forefoot between October 2022 and January 2023. In both datasets, there was a high prevalence of risk factors for delayed healing or pseudoarthrosis.

**Figure 1:**
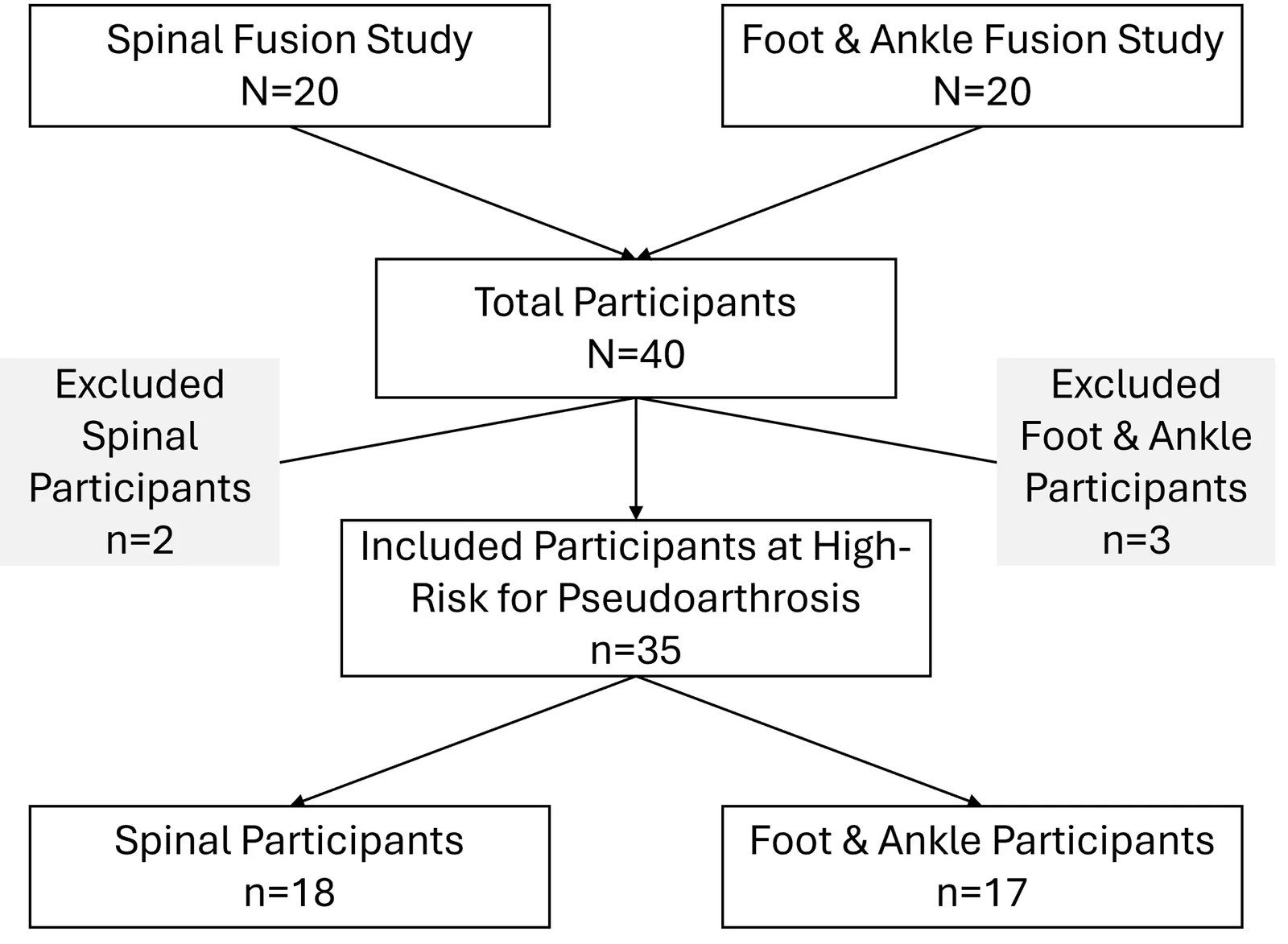
Study participant flow chart

The clinical outcomes from both studies have been published separately [20,21], which report the source data for the parent cohorts pooled in this analysis. We identified 35 participants from the combined dataset who were at risk for pseudoarthrosis from having one or more of the following risk factors: aged 65 years or older, current smoker, diabetic, obese, current steroid use, renal disease, osteoporosis, hypertension, cardiovascular disease, peripheral vascular disease, undergoing revision surgery, and/or undergoing ≥3-level spinal fusion. The five participants excluded from the parent cohorts (two spinal, three foot and ankle; see Figure 1) did not meet these risk-factor criteria. A participant flow chart is provided in Figure 1.

Endpoints

The primary efficacy endpoint was the achievement of radiographic fusion at standard timepoints. Fusion was assessed using plain radiographs at each protocol timepoint in both cohorts. Due to the retrospective nature of the study, CT was not performed routinely across the cohort but was additionally available for some participants and corroborated the radiographic evaluation results.

For spinal procedures, fusion was independently assessed by a blinded radiologist using the Bridwell interbody fusion grading system (Grades I or II were considered fused per literature definition) [22] and the Glassman posterolateral fusion grading system (Grades 4 or 5 were considered fused per literature definition) [23] at 3-, 6-, and 12-month postoperative. For foot and ankle procedures, a participant was deemed fused if 50% or more of the fusion site had osseous bridging on radiographs, per unblinded operative surgeon evaluation at 2-, 6-, 12-, and 24-week postoperative (no formal inter-observer reliability testing was performed). Safety endpoints included the incidence of major complications, graft failure, pseudoarthrosis, or unplanned surgical reintervention.

Data analysis was performed by an independent biostatistician. No a priori sample size or power calculation was performed, as this was a retrospective analysis. Demographics and clinical outcomes are displayed using means with standard deviations, medians (with the 25th and 75th quartiles), and counts with percentages. Independent samples t-tests were used to compare the number of risk factors between groups. Time to fusion was compared between groups using two-sided Mann-Whitney tests. Multivariable regression modeling of individual risk factors was not performed given the limited sample size. Comparisons between subgroups were performed without correction for multiple comparisons due to subgroup sizes. SPSS version 29 (IBM Corp.; Armonk, New York, USA) was used for analyses. p-values less than 0.05 were considered statistically significant.

## Results

Of the 35 participants included in this analysis, 34 participants had complete follow-up through their respective primary endpoint (12 months for the spinal cohort; 24 weeks for the foot and ankle cohort). Only 1 participant (in the foot and ankle cohort) did not have a 24-week follow-up radiograph; however, they were considered fused at both their previous radiographic follow-ups (6 weeks and 12 weeks).

Participant demographics and clinical characteristics

The 35 participants included in this analysis ranged in age from 38 to 79 years (mean 61.4 ± 10.2 years), with an average of 2.6 ± 1.5 risk factors per participant (Table 1). Approximately half of the cohort were males (n=19, 54.3%), and 40% (n=14) were at least 65 years of age. The two most prevalent risk factors were hypertension (n=20, 57.1%) and obesity (n=17, 48.6%, with a mean BMI of 31.5 ± 6.5 kg/m^2^). Participants on corticosteroids (n=11) and current smokers (n=5) comprised 31.4% and 14.3% of the overall cohort, respectively.

**Table 1:** Participant demographics and clinical characteristics

	Foot/ankle (n=17)	Spine (n=18)	Total (N=35)
Age (years, mean ± SD)	62.4 ± 10.0	60.5 ± 10.6	61.4 ± 10.2
Min-max	38-76	45-79	38-79
Gender, male	10 (58.8%)	9 (50.0%)	19 (54.3%)
BMI (kg/m^2^, mean ± SD)	30.5 ± 5.9	32.4 ± 7.1	31.5 ± 6.5
Race			
White	16 (94.1%)	15 (83.3%)	31 (88.6%)
Black or African American	0 (0.0%)	3 (16.7%)	3 (8.6%)
Other	1 (5.9%)	0 (0.0%)	1 (2.9%)
Ethnicity			
Non-Hispanic or Latino	16 (94.1%)	7 (38.9%)	23 (65.7%)
Hispanic or Latino	1 (5.9%)	1 (5.6%)	2 (5.7%)
Unknown	0 (0.0%)	10 (55.6%)	10 (28.6%)
High risk characteristics			
RFs per participant, mean ± SD	2.1 ± 1.0	3.1 ± 1.7	2.6 ± 1.5
Min-max	1-4	1-7	1-7
Obesity (>30 BMI)	8 (47.1%)	9 (50.0%)	17 (48.6%)
Age ≥65 years	8 (47.1%)	6 (33.3%)	14 (40.0%)
Smoker, current	3 (17.6%)	2 (11.1%)	5 (14.3%)
Steroids, current	4 (23.5%)	7 (38.9%)	11 (31.4%)
Diabetes	0 (0.0%)	1 (5.6%)	1 (2.9%)
Osteoporosis	1 (5.9%)	0 (0.0%)	1 (2.9%)
Cardiovascular disease	1 (5.9%)	6 (33.3%)	7 (20.0%)
Peripheral vascular disease	0 (0.0%)	1 (5.6%)	1 (2.9%)
Hypertension	8 (47.1%)	12 (66.7%)	20 (57.1%)
Renal disease	1 (5.9%)	4 (22.2%)	5 (14.3%)
>3 spinal levels fused	--	13 (72.2%)	--
Reoperative surgery	2 (11.8%)	7 (38.9%)	9 (25.7%)

On average, spinal fusion participants had more high-risk characteristics than foot and ankle participants (3.1 ± 1.7 vs. 2.1 ± 1.0); however, this difference was not statistically significant (p=0.060) -- a marginal result that may reflect limited statistical power in this comparison (Table 1). Seven (38.9%) participants in the spinal cohort were undergoing a reoperative procedure to repair non-union or treat adjacent segment disease, and the majority of this cohort (n=13, 72.2%) underwent fusion procedures at 3 or more levels. Systemic diseases were prevalent, including hypertension (n=12, 66.7%), cardiovascular disease (n=6, 33.3%), renal disease (n=4, 22.2%), peripheral vascular disease (n=1, 5.6%), and type 2 diabetes (n=1, 5.6%).

The foot and ankle cohort similarly consisted of high-risk characteristics. Two (11.8%) participants underwent revision repairs of previous surgeries. Comorbid conditions included hypertension (n=8, 47.1%), cardiovascular disease (n=1, 5.9%), renal disease (n=1, 5.9%), and osteoporosis (n=1, 5.9%). No participants (n=0) suffered from severe preoperative malalignment or defects.

Procedural details

Most participants in the spinal cohort (n=15/18, 83.3%) underwent fusion for multiple indications, including stenosis (n=13/15, 72.2%), herniated nucleus pulposus (n=13/15, 72.2%), spondylolisthesis (n=11/15, 61.1%), spondylosis (n=8/15, 44.4%), and reoperation (n=7/15, 38.9%), among others (e.g., scoliosis, radiculitis/radiculopathy, and kyphosis). The three remaining participants undergoing surgery for only one indication were due to stenosis (n=3/18, 16.7%). Posterolateral interbody fusion was performed on 88.9% of the spinal cohort (n=16), in part through a minimally invasive approach (n=8, 44.4% transforaminal lumbar interbody fusion or lateral lumbar interbody fusion). These patients received 10 cc of CBM within a titanium expandable interbody spacer (Globus Medical, Audubon, Pennsylvania) and 5 cc of CBM alongside posterolateral percutaneous pedicle screws. The remaining two (11.1%) participants received posterior spinal fusion with 5 cc of CBM and pedicle screws. Each participant who underwent posterior spinal fusion also received local autograft, if available.

The foot and ankle cohort consisted of participants undergoing fracture non-union repair and/or joint fusion, with at least 50% CBM used as a bone graft (if needed, crushed cancellous bone or local bone was used to extend the graft). Following forefoot and midfoot procedures (n=8/17, 47.1%), participants began partial weight-bearing in a medical boot with dressing, then returned at two weeks postoperative for suture removal. Physical therapy was usually initiated at six weeks postoperative, with participants gradually becoming full weight-bearing in the boot, then transitioning to no boot by 12 weeks. For hindfoot and ankle fusion procedures (n=9/17, 52.9%), participants stayed non-weight-bearing in a postoperative splint for the first two weeks, then a boot or cast for the following four weeks. At the six-week mark, participants advanced to touchdown weight-bearing in a boot, followed by weight-bearing as tolerated in the boot at 12 weeks, finally reaching full weight-bearing by 24 weeks.

Radiographic fusion

Despite the elevated risk profiles of included participants, the spine and foot and ankle cohorts demonstrated favorable radiographic fusion and safety outcomes in this uncontrolled series.

**Figure 2:**
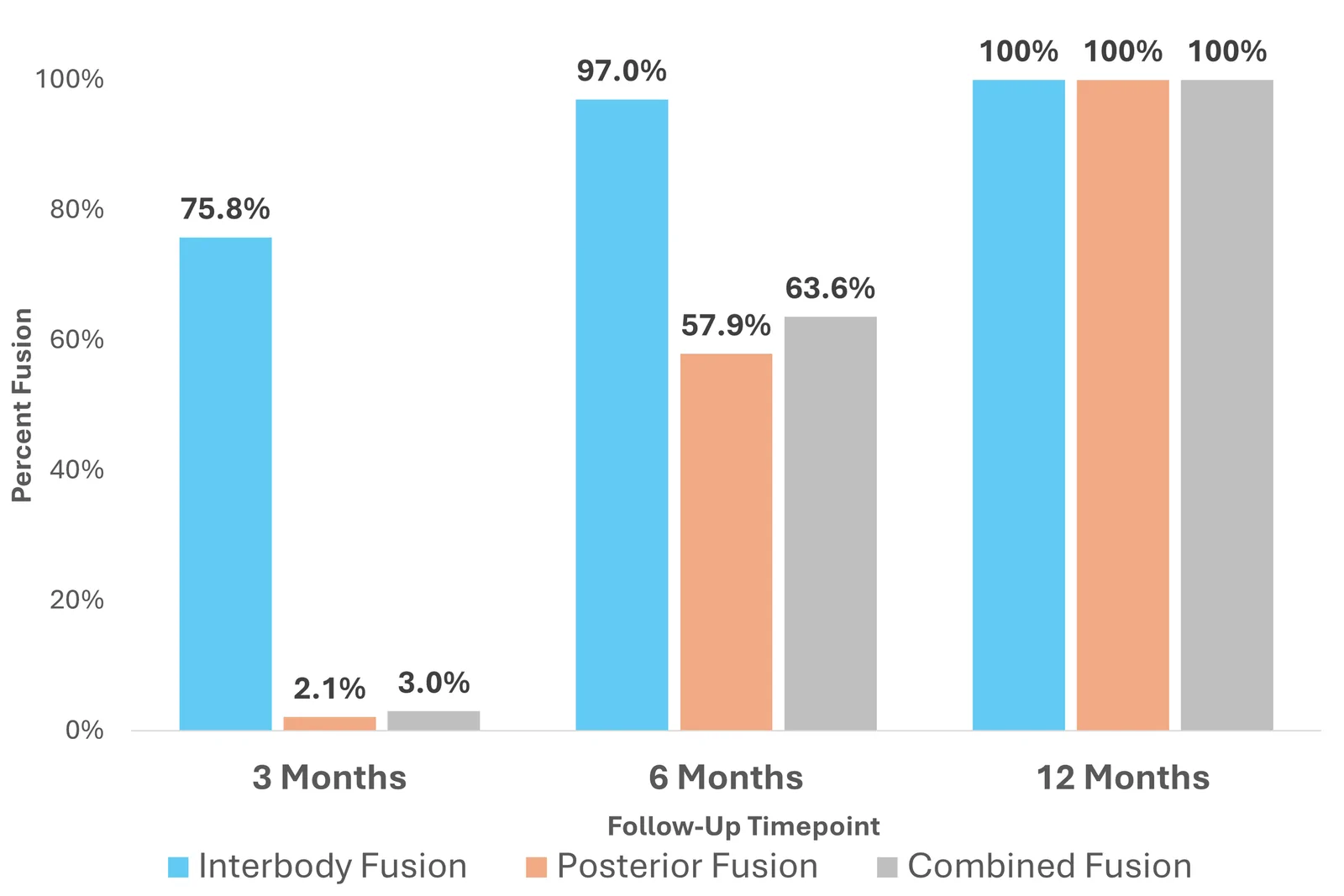
Radiographic fusion rates: spinal cohort Spinal cohort fusion rates per level over time. All levels (n=95/95, 100%) were fused by the 12-month follow-up time point.

**Figure 3:**
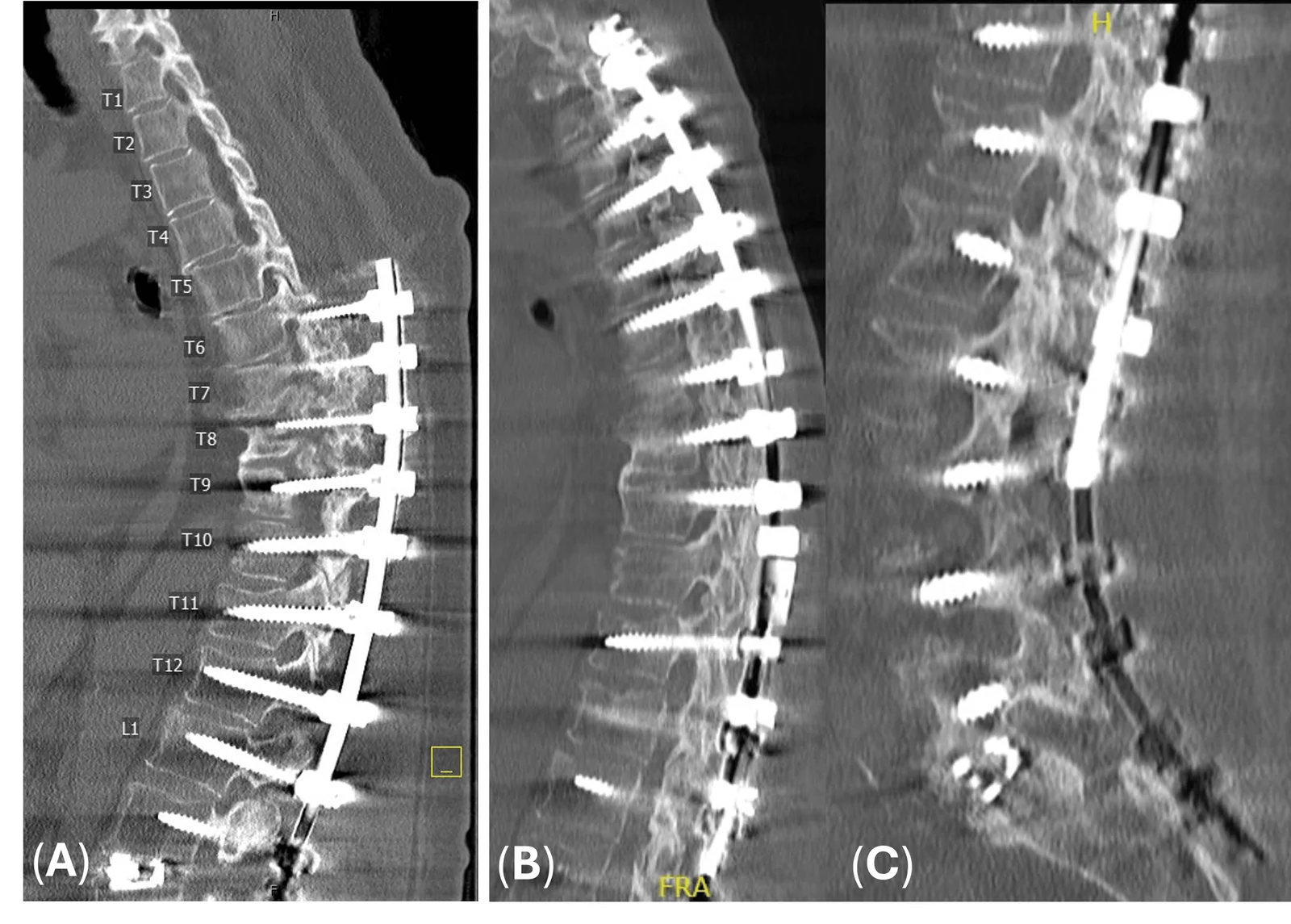
Example CT imaging from an 11-level revision posterior fusion A 61-year-old female smoker with obesity and hypertension presented with proximal junctional failure and thoracic pseudoarthrosis. Her extensive surgical history included an L3-L5 PLIF six years prior and a T6-pelvis posterior fusion with an L1-L3 TLIF eight months prior. To address the failure of the previous construct, she underwent hardware removal and a revision T2-L1 posterolateral fusion. (A) Baseline and (B, C) 12-month follow-up CTs depicting successful fusion. Image courtesy: Dr. Peter G. Passias

By 12 months, all (100%) interbody (n=33/33) and posterolateral (n=95/95) levels of the spinal fusion cohort were fused (Table 2, Figure 2). Interbody fusion rates by level progressed rapidly from 75.8% (n=25/33) at three months to 97.0% (n=32/33) at six months, with a median fusion time of 3.0 months (3.0-3.0). Posterolateral fusion levels reached 100% union by 12 months, with a median fusion time of 6.0 months (6.0-12.0). The median time to fusion for participants with treated levels receiving graft in both the interbody and posterolateral areas (n=33) was 6.0 months (6.0-12.0). Subgroup analysis revealed that construct length (short, n=12/95 vs. long segment, n=83/95) was not associated with time to fusion (short: 6.0 (6.0-6.0) months vs. long: 6.0 (6.0-12.0) months, p=0.143), though this exploratory comparison is likely underpowered given the small, short-segment subgroup. One example of CT imaging from an 11-level revision posterior fusion performed on a 61-year-old obese, hypertensive female who used tobacco is showcased in Figure 3. This participant had multiple fusion surgeries in the years prior and presented with proximal junctional failure and thoracic pseudoarthrosis.

**Table 2:** Radiographic fusion rates: spinal cohort *Combined fusion rate indicates time level achieved fusion per both measurement scales (e.g. Bridwell Grade I or II and Glassman Grade 4 or 5) for participants with interbody and posterolateral grafting.

	Interbody (n=33)	Posterior (n=95)	Combined* (n=33)
Fusion rate per level, n (%)			
3 months	25 (75.8%)	2 (2.1%)	1 (3.0%)
6 months	32 (97.0%)	55 (57.9%)	21 (63.6%)
12 months	33 (100.0%)	95 (100.0%)	33 (100.0%)
Time to fusion, months			
Median (25th-75th percentile)	3.0 (3.0-3.0)	6.0 (6.0-12.0)	6.0 (6.0-12.0)
Mean ± SD	3.7 ± 1.3	8.5 ± 3.1	8.1 ± 4.3

**Figure 4:**
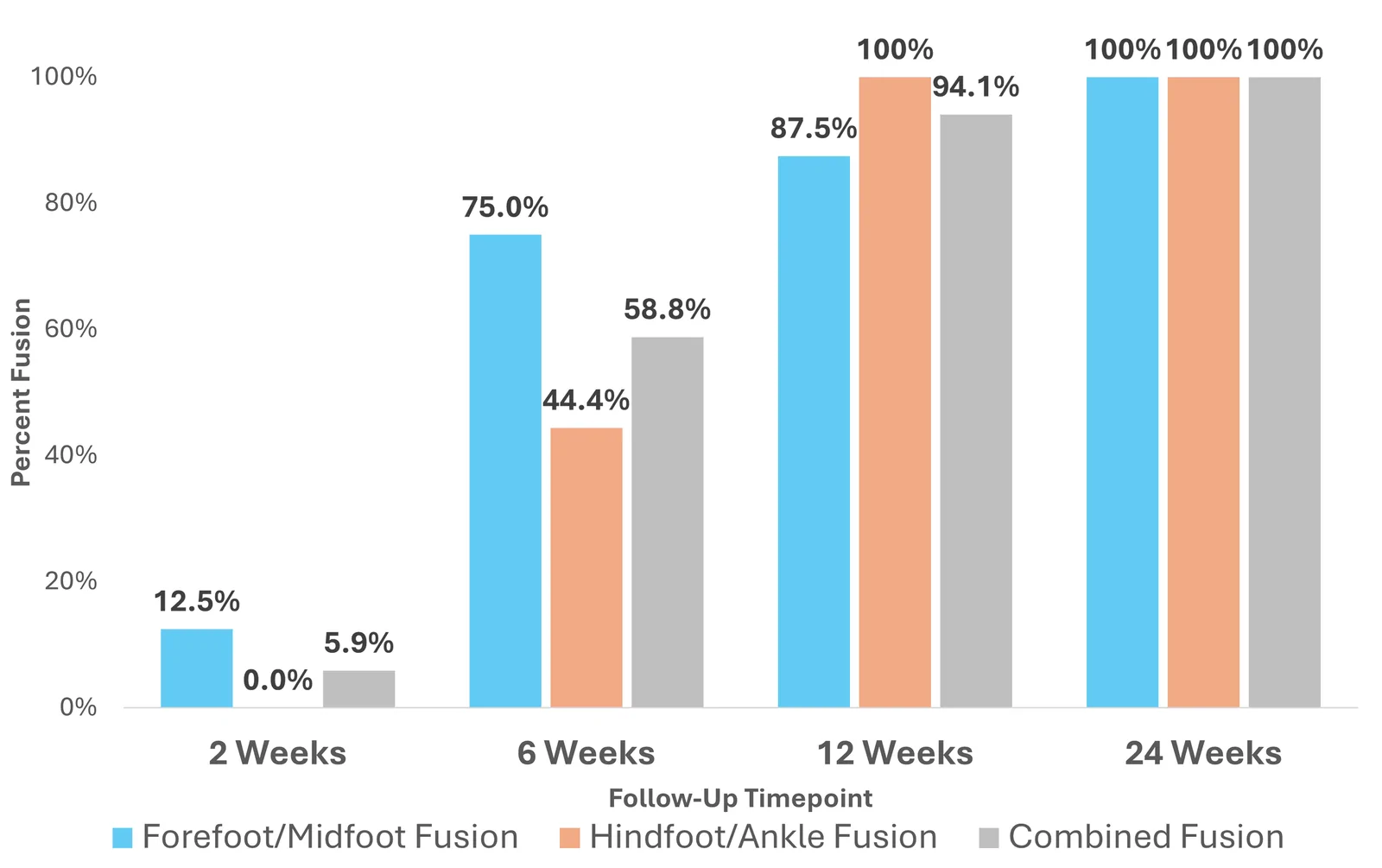
Radiographic fusion rates: foot and ankle cohort Foot and ankle cohort fusion rates per participant over time. By 12 weeks, all hindfoot/ankle participants were considered fused. All participants achieved fusion by the 24-week evaluation.

**Figure 5:**
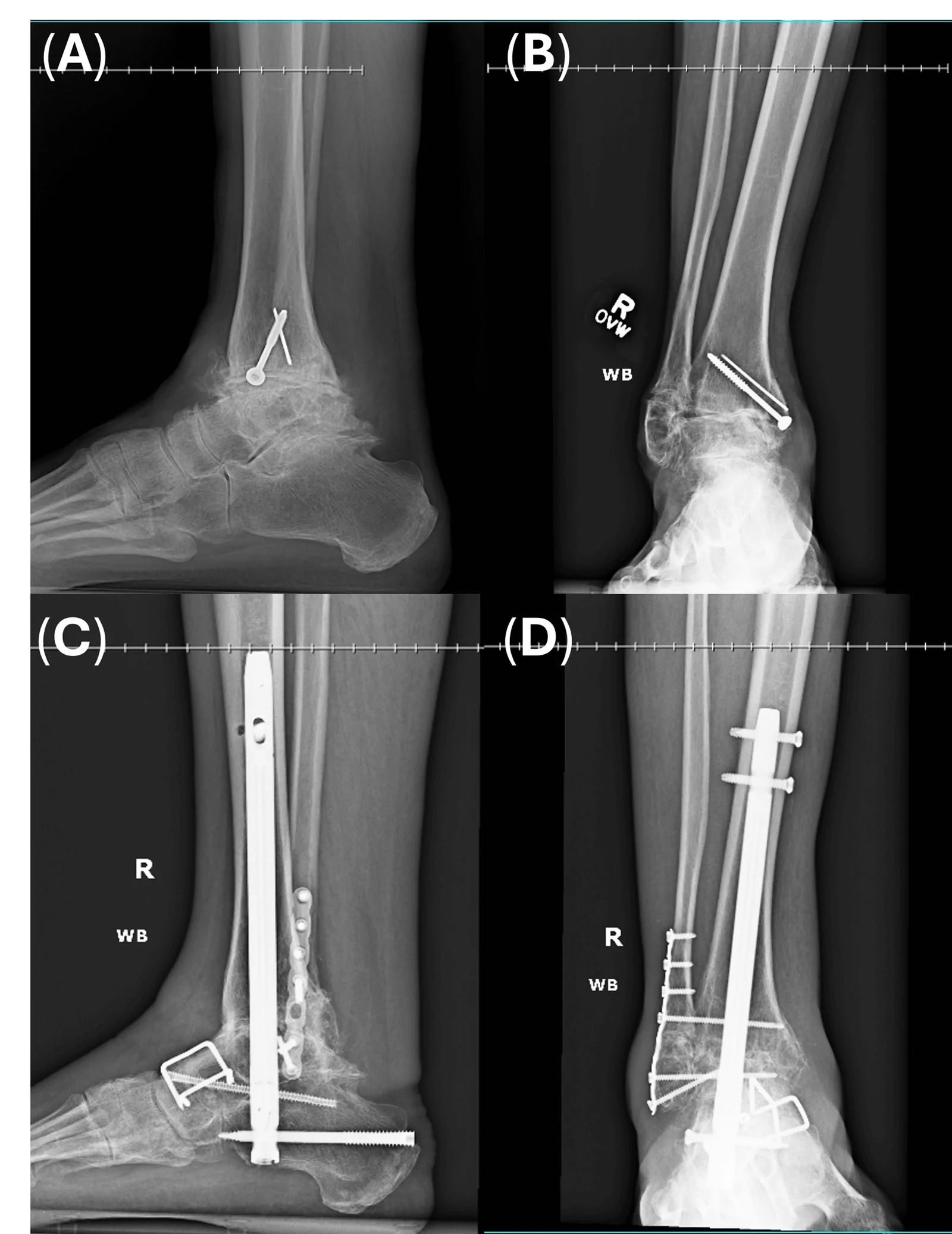
Example radiographic imaging from an ankle revision procedure (A, B) Baseline and (C, D) 20-week follow-up radiographs depicting successful fusion of a 62-year-old male participant who underwent removal of deep implanted hardware at the right ankle, a pantalar fusion, and repair of a fibular fracture non-union. Image courtesy: Dr. Kurt J. Hofmann

In the foot and ankle cohort, 58.8% (n=10/17) of participants showed radiographic union by six weeks postoperative, which increased to 94.1% (n=16/17) by 12 weeks (Table 3, Figure 4). All hindfoot/ankle participants (100%, n=9/9) achieved fusion in 12 weeks, and the overall cohort reached 100% radiographic fusion by the end of the follow-up period (24 weeks). The overall median time to fusion was 6.0 weeks (6.0-12.0), with no statistical difference between forefoot/midfoot and hindfoot/ankle participants in median weeks to fusion (6.0 (6.0-7.5) vs. 12.0 (6.0-12.0); p=0.262), though with only eight and nine participants in these subgroups, respectively, this comparison is likely underpowered. Figure 5 provides an example of ankle revision surgery in a 62-year-old male who was deemed fused per the study definition at six weeks postoperative.

**Table 3:** Radiographic fusion rates: foot and ankle cohort *One participant did not have a 24-week follow-up radiograph; however, they were considered fused at their 6-week and 12-week follow-up visits.

	Forefoot/midfoot (n=8)	Hindfoot/ankle (n=9)	Combined (n=17)
Fusion rate per participant, n (%)			
2 weeks	1 (12.5%)	0 (0.0%)	1 (5.9%)
6 weeks	6 (75.0%)	4 (44.4%)	10 (58.8%)
12 weeks	7 (87.5%)	9 (100.0%)	16 (94.1%)
24 weeks	7 (100.0%)*	9 (100.0%)	16 (100.0%)*
Time to fusion, weeks			
Median (25th-75th percentile)	6.0 (6.0-7.5)	12.0 (6.0-12.0)	6.0 (6.0-12.0)
Mean ± SD	8.5 ± 6.8	9.3 ± 3.2	8.9 ± 5.1

Safety

No perioperative or postoperative adverse events occurred in either the spine or foot and ankle cohorts that were related to the graft, and no surgical reinterventions were performed due to graft failure.

## Discussion

Arthrodesis remains a cornerstone of orthopedic surgery, yet the clinical challenge of achieving fusion in modern "high-risk" patients still exists. The demographic of patients undergoing these surgeries continues to increase toward an aging population with multiple risk factors for pseudoarthrosis, in which the local healing environment is often compromised [6,11]. Our results show that the use of a next-generation CBM achieved 100% radiographic union in at-risk participants.

Advances in regenerative medicine manufacturing have shifted industry focus toward all-in-one osteobiologic solutions that eliminate donor-site morbidity while maintaining osteoconduction, osteoinduction, and osteogenesis [24]. While common bone grafting alternatives such as synthetic scaffolds, demineralized bone matrix, and recombinant bone morphogenetic protein (BMP) offer clinical utility, they are limited by inconsistent efficacy, high lot-to-lot variability, significant safety risks, and/or a lack of one or more wound-healing components [11,24]. In contrast, next-generation CBM is manufactured using a gentle, proprietary process that preserves viable osteoprogenitor cells and high levels of bioavailable osteoinductive growth factors such as BMP-2 within the matrix to support new bone formation [11,20]. By delivering a high concentration of osteoinductive factors necessary for neovascularization and bone remodeling, we hypothesized that this novel CBM could help address potential biologic deficiencies in high-risk patients [11]; however, this mechanism was not directly assessed in the present clinical cohort, and no biologic assays were performed on the grafts used.

During bone healing, as confirmed in benchtop studies, a complex set of cytokines and growth factors coordinates tissue remodeling and neovascularization [9,11]. This includes factors such as BMPs, platelet-derived growth factor, transforming growth factor-β, vascular endothelial growth factor (VEGF), and endothelial growth factor [9,11]. For at-risk individuals, this biological environment is often compromised, which is the primary hurdle to achieving clinical success [8,11]. The presence of robust angiogenic factors (such as VEGF) is theorized to be particularly crucial for patients with comorbidities that compromise local blood supply [11]. Table 4 outlines common risk factors and their cellular and vascular consequences on the bone remodeling cycle [6,12,13,25-37].

**Table 4:** Common risk factors for pseudoarthrosis and their biological impact on fusion BMP, bone morphogenetic protein; VEGF, vascular endothelial growth factor.

Risk factor	Impact on bone healing
Tobacco use	Nicotine constricts blood flow, inhibits the development and activity of osteoblasts, and suppresses the expression of angiogenic cytokines (e.g., VEGF), which is essential for angiogenesis [37].
Diabetes	Chronic hyperglycemia impairs bone collagen synthesis and slows mineralization, leads to microvascular damage and chronic inflammation, and reduces the expression of critical bone growth factors (e.g., VEGF and BMPs) [34-36].
Osteoporosis	Increased intracortical porosity and excessive bone matrix resorption create a biomechanically weak environment for graft incorporation [12,13,33].
Corticosteroids	Chronic use leads to reduced serum calcium, which can contribute to osteoporosis, and high doses can cause avascular necrosis [6,32].
Obesity	Large amounts of adipose tissue can lead to hormonal disruptions, chronic inflammation, and poor metabolic health (e.g., diabetes), all of which can disrupt the bone healing cycle [30,31].
Vascular disease	Limited blood flow prevents nutrients and endogenous cells from reaching the fusion site for healing [29].
Hypertension	Chronic high blood pressure causes vascular damage, which can disrupt nutrient delivery, and is linked to reduced bone mineral density through secondary osteoporosis [27,28].
Renal disease	Impaired kidney function leads to osteodystrophy by disrupting the metabolic balance of calcium and phosphorus levels (leading to secondary hyperparathyroidism), and lowers vitamin D activation, all of which reduce the quality of bone [12,26].
Age (65+)	Aging leads to cellular senescence, reduced quantity and quality of mesenchymal stem cells, adipogenesis vs. osteogenesis, and decreased prevalence of osteocytes [25].

The high prevalence of multimorbidity in our study population, averaging 2.6 risk factors per participant, typically correlates with elevated rates of pseudoarthrosis, which the literature suggests can reach as high as 41% in complex reconstructions [1-3]. In our experience, the use of the novel CBM was associated with no reported instances of non-union or graft-related revisions. However, differences in study design (e.g., patient selection, surgical technique, and outcome assessment methodology) could account for some or all of the observed difference.

In the spinal cohort, nearly 40% of participants were undergoing revisions of previous failures (n=7/18), yet they achieved fusion across all operative levels by 12 months. Interestingly, time to fusion was not significantly affected by construct length as was expected, which may be an artifact of underpowering, given the small sample size of the short-segment subgroup (n=12/95 levels) [10]. Particularly noteworthy was the rapid healing observed in highly compromised individuals; for instance, a 77-year-old female with tobacco use, obesity, steroid dependency, hypertension, and vascular disease achieved radiographic fusion of an L4-L5 posterolateral level within three months. One possible explanation is that growth factors preserved within the novel CBM, such as BMPs and angiogenic proteins, support the signaling cascade needed for new bone formation even when the patient's biologic environment may be compromised. However, this mechanistic explanation is speculative but could be used to guide future preclinical and translational studies.

Similarly, the foot and ankle cohort demonstrated excellent clinical outcomes, reaching a 94.1% fusion rate by 12 weeks (n=16/17) and 100% by 24 weeks (n=16/16). One obese, hypertensive, 63-year-old female who underwent a left first metatarsophalangeal joint fusion achieved fusion per study definition at the two-week radiographic follow-up. The rate of major complications, including non-union, was 0% (n=0), differing from indirect, historically reported rates of 10%-41% [2,3]. Differences in patient selection, era, surgical technique, and outcome-assessment methodology between this study and the cited literature may account for some or all of the observed difference. While the literature often reports longer fusion times and higher non-union rates for hindfoot and ankle vs. forefoot and midfoot procedures due to increased mechanical challenges [38], our study surprisingly found no statistically significant difference in median time to fusion between the forefoot/midfoot and hindfoot/ankle subgroups. Due to the small subgroup sizes (n=8 forefoot/midfoot vs. n=9 hindfoot/ankle), this comparison is likely underpowered.

## Conclusions

These observational findings highlight the utility of this next-generation CBM in a high-risk patient population undergoing complex spine and lower extremity arthrodesis. In each cohort, we observed favorable radiographic fusion rates and no reported graft-related complications.

Despite these promising outcomes, this study has several limitations that affect interpretation of the findings. Its small retrospective post-hoc design, no a priori power analysis, non-pre-specified subgroup selection, anatomically heterogeneous population, and lack of a control group or blinding limit the ability to draw definitive conclusions or to compare with other bone graft substitutes and carry an inherent risk of selection bias. The cohort's demographic composition reflects that of the two contributing single-center institutions and may not be representative of the broader population undergoing these procedures nationally. The small size of individual risk factor subgroups within this cohort also limits interpretation of the data as demonstrating efficacy for any specific individual risk factor. Due to the different anatomic locations of the two cohorts, the parent datasets differed in fusion assessment criteria and follow-up schedules, which is why fusion rates are reported separately in this manuscript. Finally, although the dataset was analyzed by an independent biostatistician, manufacturer funding of the study could have created potential bias. Future prospective, randomized, blinded controlled trials with larger, powered patient populations and longer-term surveillance are needed to confirm these findings, assess long-term durability, evaluate causation, and determine the broader clinical impact of this next-generation bone graft.
